# Inheritable Epigenetic Memory Induced by Parental Salt Stress Influences Transgenerational Plasticity of *Phragmites australis*

**DOI:** 10.1002/ece3.73343

**Published:** 2026-03-30

**Authors:** Yu‐Han Chen, Chun‐Lin Wang, Jian‐Qiao Meng, Yi‐Fan Liu, Tao Fang, Yao‐Jun Zhu, Fang‐Li Luo

**Affiliations:** ^1^ School of Ecology and Nature Conservation Beijing Forestry University Beijing China; ^2^ College of Biological Sciences and Technology Beijing Forestry University Beijing China; ^3^ Institute of Ecological Conservation and Restoration, Research Institute of Wetland, Chinese Academy of Forestry Beijing China; ^4^ Zhanjiang National Research Station for Mangrove Wetland Ecosystem Zhanjiang Guangdong China; ^5^ The Key Laboratory of Ecological Protection in the Yellow River Basin of National Forestry and Grassland Administration Beijing China

**Keywords:** clonal parental effects, DNA methylation, epigenetic regulation, estuarine wetland, *Phragmites australis*, salt stress, transgenerational plasticity

## Abstract

Phenotypes of estuarine plants are influenced by salt stress experienced in both the current generation and by their parents, a phenomenon potentially regulated by DNA methylation. In clonal plants, DNA methylation information is effectively transmitted across generations, further influencing offspring phenotypes. However, the role of DNA methylation in clonal transgenerational plasticity and its heritable stability remains poorly understood across various genotypes of wild plants. To this end, we employed controlled genotype × environment interaction experiments to investigate phenotypic responses and DNA methylation in parental and offspring generations of *Phragmites australis* under salt stress conditions. Furthermore, we investigated the stability of DNA methylation inheritance across three generations exposed to continuous salt stress. Our results demonstrated that parental salt stress significantly increased plant height, maximum leaf area, and rhizome nodes of *P. australis* offspring from certain genotypes subjected to salt stress similar to their parents, compared to offspring of unstressed parents. Parental salt stress induced an increase in CHG hemi‐methylation and a decrease in CG methylation, potentially modulating changes in offspring plant height, maximum leaf area, and rhizome nodes. Moreover, multigenerational salt stress resulted in a persistent reduction in CG methylation and a cumulative elevation of CHG hemi‐methylation in specific genotypes. These findings reveal that offspring phenotypes in *P. australis* are jointly determined by genetic background and both parental and offspring environments, mediated through epigenetic modifications, which further advances our understanding of evolutionary adaptation strategies in clonal plants.

## Graphical abstract

Offspring phenotypes are affected by genotype, parental salt stress, and offspring salt stress. Parental salt stress can influence offspring phenotypes through associated changes in DNA methylation. Multigenerational salt stress induces cumulative inheritable epigenetic changes.

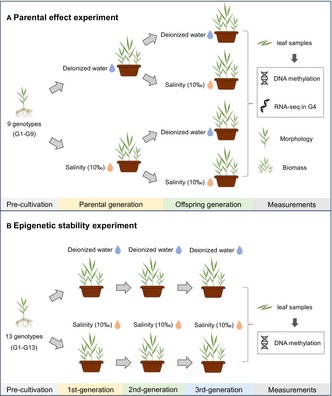

## 1 Introduction

Estuarine plants are commonly exposed to challenging environmental conditions encompassing hydrological fluctuations and salinity gradients due to cyclic tidal inundation (Bui 2013; Liu, Wu, et al. 2023). Phenotypic plasticity, defined as the ability of a given genotype to express various phenotypes under different environmental conditions, may allow plants to cope with such stress (McAndry et al. 2025). Such environments can directly influence plant phenotypes and physiological development through within‐generation plasticity (Auge et al. 2017). In addition, the environments experienced by parental plants can shape offspring performance and promote adaptive strategies under predictable environmental stimuli through transgenerational plasticity, a phenomenon commonly described as parental effects (Agrawal 2002; Dong, Alpert, et al. 2019; Waterman and Sultan 2021; Zhang et al. 2025). Parental experiences in saline environments may lead to adjustments in growth cycles, reproductive strategies, and physiological metabolism of the offspring, enabling plant adaptation to high‐salinity conditions through early germination, longer reproductive periods, and increased biomass allocations (Lin et al. 2022; Wang, Baskin, et al. 2022). However, long‐term salt stress may also constrain offspring growth and even reduce adaptive capacity (Groot et al. 2016).

Beyond nutrient provisioning and metabolite exchange, parental effects can also be mediated by heritable epigenetic regulation of offspring phenotypes through DNA methylation, histone modifications, and small RNAs, without modifying DNA sequence (Liu et al. 2022; Sammarco et al. 2025). Among these, DNA methylation is a prevalent mechanism that regulates parental effects in plants (Herman and Sultan 2016; Xue et al. 2022) and mainly occurs in CG, CHG, and CHH contexts, where H denotes A, C, or T nucleotide bases and is associated with gene expression regulation and genome stability. However, its functional role depends on genomic location (Geng et al. 2020). Generally, CG methylation is the most stable and plays a critical role in transgenerational inheritance, whereas CHG methylation is more dynamic (Brunel‐Muguet et al. 2025; Laanen et al. 2021; Platt et al. 2015). Alterations in these methylation contexts can influence phenotypic traits such as plant growth and flowering time (Lodhi and Srivastava 2025; Morgan and Donohue 2022; Shahzad et al. 2025).

Environmental stimuli can induce altered methylation patterns that persist throughout the plant's life cycle, resulting in transgenerational stress memories and leading to the transmission of adaptive environmental information across generations (Cao and Chen 2024; Talarico et al. 2024). Such memory facilitates rapid responses when the plant encounters the environmental stimulus again, contributing to the quicker adaptation of the offspring (Panda et al. 2023). For instance, multigenerational cold stress in *Oryza sativa* induced heritable hypomethylation at both CG and CHG sites within the *ACT1* promoter, conferring enhanced cold tolerance in offspring (Song et al. 2025). A plausible mechanism is that parental salt stress induces heritable DNA methylation changes that regulate targeted gene expression (Aycan et al. 2024; Tang et al. 2024; Vatov and Gechev 2025; Wibowo et al. 2016). While DNA methylation inheritance has been extensively investigated in sexually reproducing plants, its role in clonally propagating species remains poorly characterized.

Clonal plants potentially produce independent genetically identical ramets through clonal propagation (Barrett 2015). Environmental cues experienced by the parent can be transmitted between interconnected ramets via clonal propagules such as stolons and rhizomes, influencing the phenotypes of the clonal offspring, a phenomenon known as clonal parental effects (Xing et al. 2024; Zhang et al. 2022). Compared to sexual offspring, clonal offspring often experience more predictable environments. Unlike sexual reproduction, clonal propagation lacks meiosis and can bypass the resetting of epigenetic information, allowing clonal parental plants to transmit environmental information to their offspring more efficiently (Anastasiadi et al. 2021; Gonzalez et al. 2018; Yu et al. 2022). Previous research has demonstrated that clonal parental effects can influence offspring phenotypes, primarily at the species level (Dong, Alpert, et al. 2019; Quan et al. 2022). The direction and magnitude of parental effects may vary considerably among different genotypes, suggesting that the phenotype of the clonal offspring is not only influenced by the environment but may also be co‐regulated by plants' genetic and epigenetic status (Zhang et al. 2023). Nonetheless, the persistence of the epigenetic markers and their stable transmission across generations during clonal propagation remains highly variable (Groot et al. 2016; Virgen et al. 2025). Therefore, it is imperative to investigate the epigenetic regulation of transgenerational phenotypes and their heritable stability among different genotypes (Sammarco et al. 2025).

Our study determined the phenotypes and DNA methylation patterns of nine genotype groups (hereafter genotypes) of *Phragmites australis* (Cav.) Trin. ex Steud exposed to salt stress or control treatments in the parental generation, with their offspring subsequently subjected to either treatment. Differentially expressed genes (DEGs) were determined and annotated to identify gene pathways associated with transgenerational responses under parental salt stress. Thirteen genotypes were further selected to assess the stability of inheritance of DNA methylation patterns in three consecutive generations under control and salt stress conditions. This study specifically addressed the following three questions: (1) Does parental salt stress induce adaptive phenotypes and alter DNA methylation in offspring of different *P. australis* genotypes when re‐exposed to salt stress? (2) How are offspring DNA methylation patterns associated with transgenerational phenotypic responses to salt stress, and which gene pathways are involved? (3) Is the persistence of environmentally induced DNA methylation changes maintained across generations under continued salt stress?

## 2 Materials and Methods

### 2.1 Study Material

*P. australis* is a perennial herb commonly found in rivers, lakes, ponds, and ditches along the shoreline and low wetlands worldwide in temperate, subtropical, and tropical areas. It has well‐developed rhizomes and reproduces both clonally and sexually. Ecotypic and genotypic differentiation is pronounced, with strong nutrient utilization, photosynthesis, and clonal propagation (Fant et al. 2016; Wang et al. 2021). As a dominant species in estuarine wetlands, *P. australis* frequently experiences fluctuating salinity conditions and plays an important role in coastal ecosystem functioning (Song et al. 2024). Its high genetic diversity, pronounced phenotypic plasticity, and strong clonal growth across salinity gradients make it an ideal model for investigating transgenerational responses and epigenetic mechanisms underlying plant adaptation to salt stress.

In this study, a Neighbor‐Joining phylogenetic tree was constructed using Simple Sequence Repeat (SSR) markers for 416 *P. australis* individuals from the Liaohe, Yellow River, Yangtze, and Minjiang River estuaries in China. These individuals were classified into 25 genotype groups (Figure S1), following the sampling and SSR protocols described in Chen et al. (2024). From these groups, a total of 13 genetically distinct and robust genotypes (G1–G13) were selected as the experimental material. Two complementary experiments were conducted to investigate phenotypic and epigenetic responses of *P. australis* to salt stress across generations. The first experiment employed nine genotypes (G1–G9) and examined parental effects and transgenerational interactions across two clonal generations (parental and offspring generations). The second experiment utilized all 13 genotypes (G1–G13) to assess the stability of DNA methylation patterns across three consecutive generations grown under either control or salt stress conditions. In the parental effect experiment, genotype G4 was additionally selected for gene expression analysis due to its consistent growth performance and availability of sufficient and uniform leaf material suitable for RNA extraction. The rhizomes of the selected plants were cultivated in a greenhouse for nearly one year at the Beijing Shengfang Technology Co. Ltd., China (40°0′27″ N, 116°20′19″ E) to minimize variation induced by the originating habitat. During the two experimental periods, the average greenhouse temperature and relative humidity were maintained at 18.72°C and 60.43%, respectively.

### 2.2 Parental Effects of *P. australis* Plants Grown Under Salt Stress

**FIGURE 1:**
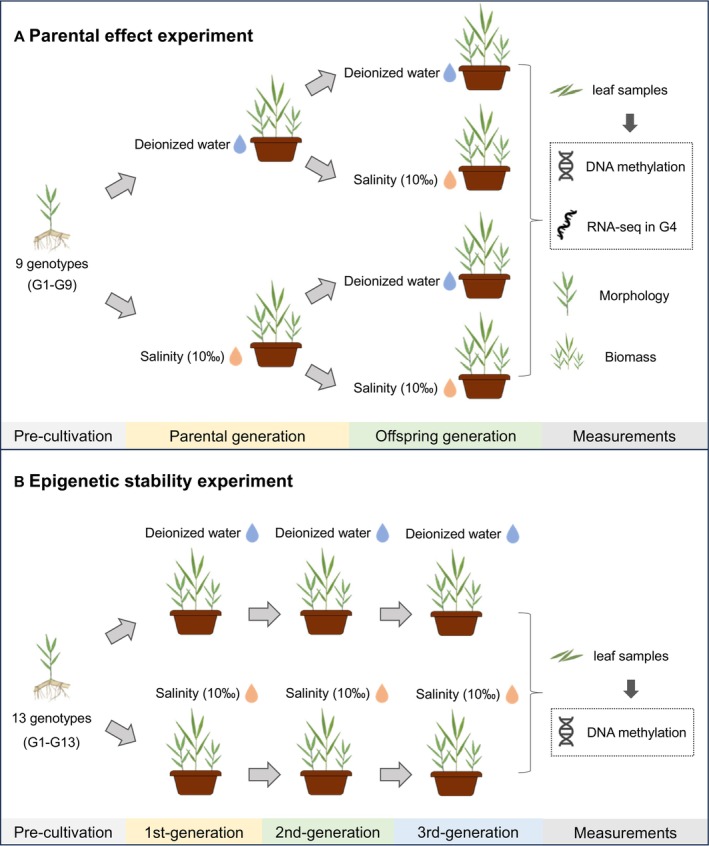
Experimental design for the evaluation of the impact of parental salt stress on progeny traits (A) and transgenerational epigenetic stability (B).

The experiment consisted of two generations and spanned from August 2021 to October 2022 in a greenhouse. For the parental generation, uniformly growing *P. australis* seedlings, each with a 10 cm rhizome and an approximate height of 50 cm, were selected and transplanted into individual perforated pots (17 cm in diameter and 14 cm in height) filled with a 1:1 (v/v) substrate mixture of washed river sand and clay loam (sourced from a commercial nursery in Beijing). Each perforated pot was then placed inside a larger non‐perforated pot (21 cm in diameter and 18 cm in height), serving as a reservoir for deionized water or salt solutions. The salt stress experiment for the parental generation commenced in early August 2021 and lasted for four months, until early December 2021. The control treatment consisted of deionized water with 0‰ salt, while the salt stress treatment was conducted using a 10‰ sea salt solution, representative of moderate salinity within the natural estuarine range of *P. australis* (Achenbach and Brix 2014; Li et al. 2020). Six replicates were established for each of the nine genotypes (G1–G9; Figures 1A, S1), resulting in a total of 108 parental plants (9 genotypes × 2 treatments × 6 replicates). The water lost to evaporation was replenished periodically during the experiment by maintaining a constant water level 1 cm above the soil surface. Growth traits were measured, and plants were harvested in early December 2021.

For the offspring generation, 10 cm rhizome tips derived from parental plants (parental generation) were used to establish progeny populations for the assessment of transgenerational interactions. Due to low temperatures in winter (4°C–12°C), rhizomes sprouted very slowly. To mimic a natural overwintering phase, the offspring required a prolonged period to reach an average height of about 50 cm. Therefore, the control and salt stress treatments for the offspring were initiated in mid‐June 2022 and lasted for four months until mid‐October 2022. The treatments in the salt stress experiment to assess interactions in the offspring consisted of parental and offspring control (CC), parental and offspring salt stress (SS), parental control and offspring salt stress (CS), and parental salt stress and offspring control (SC), with five replicates for each of the nine genotypes (G1–G9; Figures 1A, S1), resulting in a total of 180 offspring plants (9 genotypes × 4 treatments × 5 replicates). The pots, substrate, watering setup, and salt concentration used in the offspring experiment were identical to those used in the parental line experiment. Phenotypic indicators were subsequently measured, and plants were harvested in mid‐October 2022.

### 2.3 Epigenetic Stability Across Generations Under Continued Salt Stress

To assess the persistence of environmentally induced DNA methylation patterns across generations, a separate experiment was conducted using three consecutive clonal generations. First generation (F1): Uniformly grown *P. australis* seedlings, each with a 10 cm rhizome and an average height of approximately 50 cm, were transplanted into individual pots using the same potting system, substrate mixture, and salt stress treatment setup as described for the parental effect experiment. The first‐generation experiment commenced in early July 2021, with two treatments: a control treatment with deionized water and a salt stress treatment with 10‰ sea salt solution, applied across 13 genotypes with six replicates each (G1–G13; Figure 1B and Figure S1), resulting in a total of 156 plants (13 genotypes × 2 treatments × 6 replicates). Plants were harvested and measured at the end of October 2021, after approximately four months of salt stress exposure for first‐generation plants.

Second and third generations (F2 and F3): Newly generated 10 cm rhizome tips from the preceding generation were used to establish subsequent offspring plants. After most rhizomes had sprouted, salt stress treatments for each generation were applied from late January to October 2022 and 2023, each lasting nine months. Each treatment included six replicates, with 156 plants per generation (13 genotypes × 2 treatments × 6 replicates). The materials, conditions, and timing of trait measurements remained consistent across generations. The experiment to assess epigenetic stability lasted from July 2021 to October 2023.

### 2.4 Measurements

To evaluate plant growth performance and potential adaptive responses to salt stress, several morphological and growth traits associated with structural growth, resource acquisition, and clonal propagation were measured. In the experiment assessing parental effects, the maximum leaf area of the physically tallest ramet was determined using a LI‐3000C portable leaf area meter (LI‐COR, USA). Plant height and basal stem diameter were measured for the physically tallest ramet, and the total node number of underground rhizomes was counted in each pot. Fresh weight was determined for both 10 cm rhizome segments and the remaining rhizome tissue to calculate the water content.

At harvest, leaves of plants across all experiments were also collected at the end of each stress treatment period. From each pot, three healthy, fully expanded young leaves were randomly selected, weighed fresh, then dried with silica gel for subsequent DNA extraction and leaf water content determination. Subsequently, the above‐ground and the remaining underground plant tissues were dried in an oven at 70°C for 72 h and then weighed to obtain the corresponding dry biomass.

DNA extraction and methylation‐sensitive amplified polymorphism (MSAP) analyses were performed as described previously (Chen et al. 2024). Briefly, genomic DNA was digested with the methylation‐sensitive restriction enzymes HpaII and MspI in combination with EcoRI, followed by adaptor ligation, pre‐selective, and selective PCR amplification. The final selective amplification products were sent to Sangon Biotech Company (Shanghai, China) for capillary electrophoresis detection and scored as binary data. The methylation status at each locus was inferred from the presence/absence patterns across the two enzyme combinations. Four biological replicates were analyzed for each genotype under different treatments due to tissue availability and logistical constraints associated with molecular analyses.

### 2.5 Sequencing

To assess gene expression changes caused by parental salt stress, we collected additional fresh leaf samples from offspring G4 plants subjected to salt stress in the parental effect experiment, whose parental lines had undergone different treatments (CS and SS; three replicates each). We conducted transcriptome analyses to assess their increased tolerance to salt stress. Leaves were sampled at the beginning of harvest from the same experimental pots used for MSAP analyses. From each pot, three healthy, fully expanded young leaves were randomly selected, and their fresh weight was measured for the subsequent determination of dry biomass. The selected leaves were then rinsed with sterile water and disinfected with 75% alcohol, subsequently sectioned into 0.5 cm fragments, and then rapidly frozen in liquid nitrogen before storage at −80°C. Leaf RNA extraction and data analysis were performed by Novogene (Beijing, China). The total RNA was extracted with the Polysaccharide Polyphenol Plant Total RNA Extraction Kit (QIAGEN, Germany), and the quality was assessed using the Agilent 2100 Bioanalyzer. The transcriptome sequencing library was prepared with the NEBNext UltraTM RNA Library Prep Kit for Illumina (NEB, USA). Subsequent to library construction, quantification and quality control were performed using the Qubit 2.0 Fluorometer and qRT‐PCR. Libraries that passed QC were then sequenced with the Illumina NovaSeq platform to generate 150 bp paired‐end reads. After using FASTP (version 0.19.7) for data quality control, clean reads were aligned and compared to the reference genome using HISAT2 v2.0.5, and gene expression levels (FPKM) were calculated using featureCounts v1.5.0‐p3. DEGs were analyzed using DESeq2 v1.20.0, applying a screening criterion of adjusted *p* ≤ 0.05. Gene Ontology (GO) and Kyoto Encyclopedia of Genes and Genomes (KEGG) pathway enrichment analyses were performed using clusterProfiler v3.8.1 (Yu et al. 2012).

### 2.6 Data Analyses

Data analysis and graphical visualization were performed using R 4.4.2 (R Core Team 2024). DNA methylation states, including HPA^+^/MSP^+^ (unmethylated), HPA^+^/MSP^−^ (hemi‐methylation), HPA^−^/MSP^+^ (internal cytosine methylation), and HPA^−^/MSP^−^ (full methylation or absence of target), were initially classified using the *msap* package (Guarino et al. 2019; Perez‐Figueroa 2013). For subsequent analyses, we focused on the HPA^+^/MSP^−^ (CHG hemi‐methylation) and HPA^−^/MSP^+^ (CG methylation) states, as these provide clear and unambiguous methylation information, whereas the HPA^−^/MSP^−^ state is generally considered uninformative and cannot be reliably interpreted (Perez‐Figueroa 2013). Since the MSAP analysis does not identify methylation within the CHH context, CHH methylation was not assessed.

For the parental data, the *lme4* and *lmerTest* packages (Bates et al. 2015; Kuznetsova et al. 2017) were employed to determine the effects of salt stress on phenotypic traits (height, basal stem diameter, maximum leaf area, rhizome nodes, and total biomass) and methylation levels (CHG hemi‐methylation and CG methylation) using linear mixed‐effect models (LMMs). The genotype (G), salinity environment (E), and their interactions (G × E) were treated as fixed factors, while the parental blocks were considered random factors. The rhizome nodes were analyzed using a generalized linear mixed‐effect model (GLMM) based on a negative binomial distribution due to overdispersion (residual deviance/residual degrees of freedom > 1.2). Residuals were assessed for normality and homogeneity of variance, and those that did not conform to a normal distribution were log‐transformed except for rhizome nodes.

For the offspring data, LMMs were employed for the analysis of phenotypic traits and methylation levels, and a GLMM was employed for the rhizome node data. The fixed factors included genotype (G), parental salt stress environment (PE), offspring salt stress environment (OE), and their interactions (G × PE × OE). Offspring blocks were treated as random effects, while the initial weight of offspring plants was included as a covariate for phenotypic traits. Before analysis, CHG hemi‐methylation levels, CG methylation levels, and maximum leaf area data were log‐transformed, while plant height and total biomass data were sqrt‐transformed.

For the stability experiment, the effects of genotype and generation on DNA methylation levels in continuous control and salt stress conditions were also assessed using LMMs, with genotype, generation, and their interactions treated as fixed factors and blocks as random effects. CHG hemi‐methylation and CG methylation data were log‐transformed prior to analysis.

The significance of fixed effects in all mixed‐effects models was evaluated using Type III ANOVA, employing F‐tests for LMMs and *χ* ^2^‐tests for GLMMs. Estimated marginal means for each genotype, treatment, and generation combination were extracted using the *emmeans* package (Lenth 2025). Post hoc comparisons were then performed with a Sidak adjustment, and effect sizes (treatment minus control) were calculated. Pearson correlation analysis was implemented to construct correlation matrices between offspring phenotypic traits and offspring methylation measures in the parental effect experiment, utilizing the *Hmisc* package (Harrell Jr 2025). The data were visualized using the *ggplot2* package (Wickham 2016).

## 3 Results

### 3.1 Effects of Parental Salt Stress on the Phenotype and Methylation Levels of Different Genotypes

**FIGURE 2:**
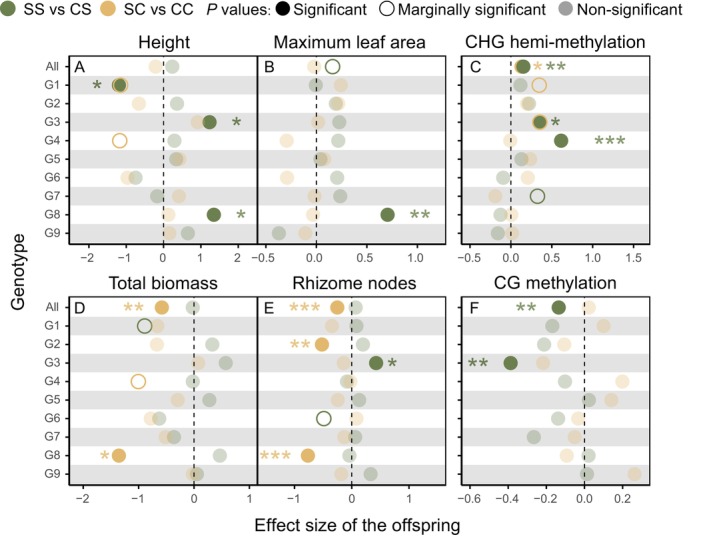
Effect size of different *P. australis* genotypes on offspring traits under control and salt stress environments following exposure to salinity or control environments in the parental generation. SS corresponds to salt stress treatments in both parental and offspring generations; CS corresponds to control treatment in the parental generation and salt stress treatment in the offspring generation; SC corresponds to salt stress treatment in the parental generation and control treatment in the offspring generation; CC corresponds to control treatments in both the parental and offspring generations. Effect sizes (illustrated by green and yellow dots) were determined based on the increase (positive) or decrease (negative) in offspring trait values in SS relative to CS or SC relative to CC. Accordingly, effect sizes positioned in the positive range indicate improved offspring trait performance following exposure to parental salt stress, while negative values reflect diminished performance after parental salt stress exposure. Asterisks indicate statistically significant effects. ***p < 0.001, **p < 0.01, *p < 0.05.

Genotypic variation in *P. australis* genotypes exerted a significant influence on CHG hemi‐methylation and CG methylation levels in the leaves. Significant differences were detected in plant height, basal stem diameter, maximum leaf area, rhizome nodes, and total biomass (Table 1). Salt stress significantly reduced plant height, rhizome nodes, and total biomass (Figure S2). In the offspring generation, CHG hemi‐methylation and total biomass were affected by the parental environment, while CG methylation and plant height were affected by genotype × parental environment interactions (Table 1). The offspring environment significantly affected plant height, basal stem diameter, maximum leaf area, rhizome nodes, and total biomass of *P. australis* . At the same time, offspring biomass and CG methylation were substantially influenced by parental × offspring environment interactions, with additional genotype‐dependent effects observed for rhizome nodes (Table 1). In offspring grown under salt‐stressed conditions, progeny derived from salt‐stressed parental lines exhibited elevated CHG hemi‐methylation levels and increased maximum leaf area but reduced CG methylation levels (Figure 2C,F). In contrast, offspring grown under control conditions, originating from parents grown under salt stress, displayed reduced biomass and fewer rhizome nodes (Figure 2D,E).

**TABLE 1:** Effects of different *Phragmites australis* genotypes [G], parental generations (P) salinity environment [(P)E], and their interaction [G × (P)E] on parental phenotypes and DNA methylation levels, and the effects of [G], parental generations environment [PE], offspring generations environment [OE], and their interactions [G × PE × OE] on offspring phenotype and DNA methylation levels.

	Traits	G	(P)E	G × (P)E	OE	PE × OE	G × PE × OE
Parental	CHG hemi‐methylation	3.95***	2.07	0.81	—	—	—
	CG methylation	3.99**	0.05	1.34	—	—	—
	Height	3.60**	25.73***	1.43	—	—	—
	Basal stem diameter	10.14***	1.06	1.59	—	—	—
	Maximum leaf area	9.65***	1.88	0.99	—	—	—
	Rhizome nodes	22.72**	4.89*	9.85	—	—	—
	Total biomass	4.75***	7.41**	0.39	—	—	—
Offspring	CHG hemi‐methylation	10.50***	14.05***	1.77	0.14	0.10	2.03
	CG methylation	1.98	3.13	2.09*	0.57	6.12*	0.42
	Height	20.03***	0.01	3.39**	94.22***	2.66	0.69
	Basal stem diameter	1.09	0.40	1.22	50.59***	3.02	1.30
	Maximum leaf area	0.39	2.01	1.22	98.51***	3.32	1.43
	Rhizome nodes	25.30**	1.82	14.03	115.74***	1.84	17.78*
	Total biomass	3.46**	6.85*	1.15	7.11**	5.63*	0.85

*Note:* *Chi‐square* values are provided for rhizome nodes, while *F* values are provided for all other traits. Appropriate denominator degrees of freedom (df) were determined by Satterthwaite's approximation. The df for parental salinity environment effects were approximately 1, 90 in the parental generation and 1, 108 in the offspring generation and offspring environment, and its interaction with the parental environment. The df values for genotype effects and their interactions with salinity were approximately 8, 90 in the parental generation and 8, 108 in the offspring generation. The df values for CHG hemi‐ and CG methylation regarding the effects of G, E, G × E were (8, 54), (1, 54), and (8, 54), respectively, in the parental generation and (8, 96), (1, 96), (1, 96), (8, 96), (1, 96), and (8, 96) for G, PE, OE, G × PE, PE × OE, and G × PE × OE, respectively, in the offspring generation. Significant effects are in bold, with *p*‐values as follows: ***p < 0.001, **0.001 ≤ *p* < 0.01 and *0.01 ≤ *p* < 0.05.

### 3.2 Mechanisms Regulating the Responses of *P. australis* to Parental Salt Stress

**FIGURE 3:**
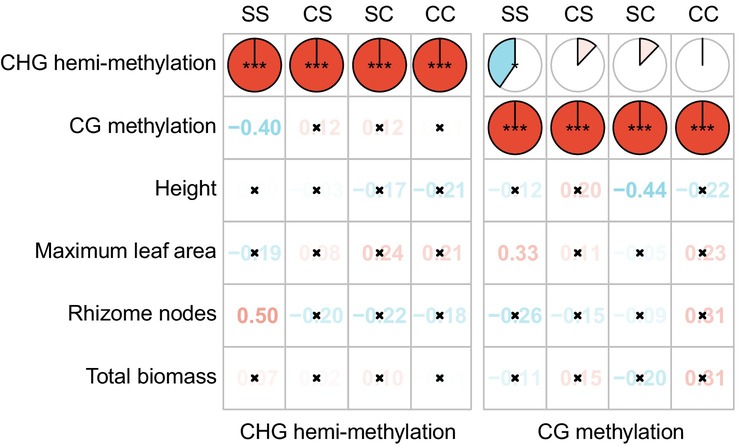
Correlation between DNA methylation levels and phenotypes in *P. australis* offspring grown under control and salt stress conditions, derived from parental plants exposed to either salinity or control conditions. Values represent Pearson's correlation coefficients, red numbers indicate significant positive correlations, blue numbers indicate significant negative correlations, and crosses indicate insignificant correlations. Comparisons between SS (salt stress in both parental and offspring generations) and CS (control in parental generation and salt stress in offspring generation), as well as between SC (salt stress in parental generation and control in offspring generation) and CC (control in both parental and offspring generations), illustrate changes in the relationships between offspring phenotypic traits and DNA methylation levels caused by parental salt stress.

Parental exposure to salinity altered the relationship between DNA methylation levels and phenotypic traits in offspring grown under both control and salt stress conditions (Figure 3). In particular, in offspring grown under salt stress conditions, parental salt stress promoted CHG hemi‐methylation and increased rhizome nodes. Moreover, the maximum leaf area was positively affected by CG methylation levels compared to *P. australis* plants originating from parental plants grown under control conditions. In offspring grown in control environments, parental salt stress experience resulted in CG methylation levels being associated with reduced plant height. Moreover, the GO functional analysis of DEGs in G4, obtained from the SS versus CS comparison in offspring grown under salt stress, showed significant enrichment of key genes involved in regulating salt stress in iron ion binding, oxidoreductase activity, tetrapyrrole binding, and heme binding (Figure S3). On the other hand, KEGG analysis of genotype G4 revealed a significant enrichment of the tryptophan metabolic pathway in offspring after experiencing parental salt stress (Figure S3). As transcriptomic analyses were conducted only for genotype G4, these enrichment results reflect responses in this genotype and may not represent all *P. australis* genotypes.

### 3.3 Generational Stability of Methylation Patterns Under Salt Stress

**FIGURE 4:**
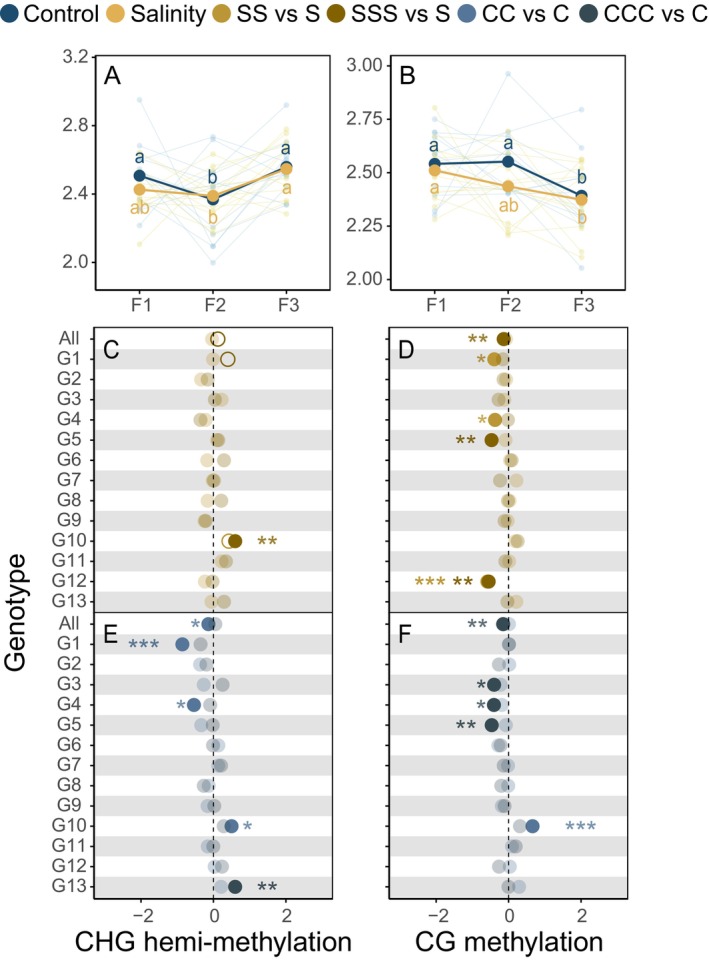
Performance of *P. australis* under control and salt stress treatments across successive generations (A, B), and effect sizes (C–F) of methylation responses of different genotypes after two or three successive generations in the control or salt stress environments. F1: First generation, F2: Second generation, F3: Third generation. S: Salt stress, C: Control. The number of S and C in the legend represents generations under salt stress or control treatments. Different lowercase letters (a and b) indicate significant differences between generations under control (blue) and salt stress (yellow) treatments. Effect sizes were calculated as the increase (positive) or decrease (negative) of the former (SS/SSS/CC/CCC) relative to the latter (S/S/C/C). For example, the effect size of SS vs. S represents the increase or decrease in methylation levels measured after two successive generations grown under salt stress relative to a single generation grown under salt stress.

Genotypic variation significantly affected CG methylation levels in *P. australis* under control and salt stress conditions (Table S1). Across generations in the control environment, CHG hemi‐methylation and CG methylation levels exhibited significant differences (Table S1). However, no linear trends regarding changes were observed between generations (Figure 4A,B), indicating fluctuating patterns. Under sustained salt stress, both CHG hemi‐methylation and CG methylation levels exhibited significant differences between generations (Table S1). Notably, CG methylation levels showed a progressive decline across generations (Figure 4B). Genotypes, generations, and their interactions had a significant impact on CHG hemi‐methylation and CG methylation levels under control environments, as well as CG methylation levels under salt stress (Table S1), suggesting differences in the genetic regulation and environmental adaptation in different *P. australis* genotypes (Figure 4C–F).

## 4 Discussion

### 4.1 Transgenerational Plasticity Induced by Parental Effects in Salt‐Stressed Environments

Salt stress significantly inhibited the growth of *P. australis* , reducing plant height, limiting rhizome proliferation, and decreasing biomass accumulation (Figure S2), consistent with many previous studies on salinity‐induced growth suppression in plants (Bui 2013; Hualpa‐Ramirez et al. 2024; Lagiotis et al. 2023). Importantly, our study also found that parental exposure to salt stress resulted in a significant reduction in biomass and rhizome nodes of offspring grown under favorable conditions (Figure 2D,E), indicating that the legacy of parental salt stress can persist into subsequent generations, even in the absence of direct stress exposure in the offspring. This phenomenon may be due to reduced resource provisioning by stressed parents, which constrains offspring growth and development, even in favorable environments (Dong, Meng, et al. 2019). Parental provisioning plays a crucial role in the establishment and initial growth of offspring, particularly in clonal plants (Wang, Si, et al. 2022).

Interestingly, our study demonstrated that parental exposure to salt stress does not always result in negative effects on *P. australis* offspring. Offspring derived from salt‐stressed parents exhibited comparable or even enhanced growth under salinity with coordinated structural adjustments, such as larger maximum leaf area, sustained biomass accumulation, plant height, and rhizome architecture (Figure 2). Similar transgenerational benefits have been documented in *A. thaliana* and *Atriplex centralasiatica* (Suter and Widmer 2013; Wang, Baskin, et al. 2022), suggesting that transgenerational stress memory allows parents to pass pre‐adaptive information to their offspring, thereby enhancing offspring tolerance to recurrent or similar stress (Aycan et al. 2024). Such transgenerational benefits tend to be particularly pronounced when offspring encounter the same environmental conditions as their parents, especially unfavorable ones (Minden et al. 2025), which may be further strengthened under resource‐limited parental environments (Liu, Man, et al. 2023).

Additionally, the differences in growth inhibition among different genotypes indicate that *P. australis* growth performance under salt stress is tightly associated with its genetic background (Table 1; Figure S2). Offspring responses to salt stress also varied among genotypes, and both the direction and magnitude of transgenerational plasticity were genotype‐dependent (Figure 2), consistent with findings in *A. thaliana* (Latzel et al. 2023; Lin et al. 2024). Notably, genotypes G3 and G8 showed prominent phenotypic adaptations under transgenerational salt stress (Figure 2A,B,E). Given increasing salinity and increasing environmental fluctuations in estuarine wetlands, these adaptive genotypes may hold particular ecological importance in such shifting environments. In addition, higher genetic diversity provides a broader set of response options for populations under stress, enhancing their stability in heterogeneous saline environments.

### 4.2 Epigenetic Memory Transmission Pathways of Parental Salt Stress Effects

Our results provide evidence that DNA methylation is dynamically modulated under salt stress and is associated with transgenerational plasticity of *P. australis* (Figures 2C,F, 3). The dynamic changes in DNA methylation levels caused by salt stress in the plant genome commonly involve cytosine methylation across CG, CHG, and CHH contexts (Qiao et al. 2025). In this study, genotypic background exerted a stronger influence on CG and CHG methylation than direct salt stress stimuli (Table 1), consistent with observations in *A. thaliana* , where genome‐wide methylation patterns are largely genotype dependent, with salt stress inducing relatively minor changes (Lin et al. 2022). However, parental salt stress can strongly induce the remodeling of DNA methylation in offspring, especially under consecutive generational exposure, leading to increased CHG hemi‐methylation and reduced CG methylation levels (Figure 2C,F). Notably, DNA methylation responses to salt stress are highly species‐specific, varying distinctly across tissue types and genomic contexts, including transposable elements (TEs), promoters, and coding regions (Konate et al. 2018; Srikant and Drost 2021; Yaish et al. 2018). For example, salt stress has been reported to increase the overall DNA methylation levels in *Olea europaea* and *Medicago truncatula* , whereas reductions in DNA methylation levels have been reported in *Glycine max* and *Thlaspi arvense* (Geng et al. 2020; Yung et al. 2024). In *A. thaliana* , salt stress‐induced CG methylation changes are predominantly localized in exons and intergenic regions, whereas differential CHG methylation occurs predominantly on TEs. These findings indicate that CGs are primarily responsible for regulating gene expression, while CHG methylation contributes mainly to the silencing of TEs (Lin et al. 2022).

In addition, our results indicate that in *P. australis* , parental salt stress‐induced methylation changes were associated with offspring plant height, maximum leaf area, and rhizome nodes (Figure 3). This observation is consistent with a previous study in the same species, which showed that artificial demethylation using 5‐azacytidine enhanced salt tolerance in inland *P. australis* populations, specifically leading to increased plant height, basal stems, and biomass under salinity stress (Song et al. 2022). Concomitant with these phenotypic changes, significant differential gene expression was observed in metabolic pathways. Transcriptomic analyses conducted for genotype G4 revealed DEGs in *P. australis* offspring derived from salt‐stressed parents compared to offspring of non‐stressed parents under salt stress. Notably, DEGs were significantly enriched in functional categories such as iron ion binding, oxidoreductase activity, tetrapyrrole binding, and heme binding (Figure S3), suggesting that salt stress memory mitigates oxidative stress, maintains cellular homeostasis, and protects the photosynthetic system by regulating photosynthesis, metal ion dynamic homeostasis, and antioxidant enzyme activities (Liu et al. 2022). Moreover, the significant enrichment in tryptophan metabolic pathways in *P. australis* offspring induced by parental salt stress exposure suggests that salt stress memory might regulate tryptophan metabolism, thereby modulating auxin synthesis and antioxidant defense mechanisms. This subsequently enhances plant stress resistance, collectively establishing the metabolic basis for transgenerational salt adaptation (Aycan et al. 2024). Collectively, the observed epigenetic modifications and differential gene expression likely act synergistically to enhance the transgenerational adaptation of *P. australis* to salinity stress. In contrast to the gradual pace of genetic evolution, this rapid transgenerational plasticity may provide an efficient adaptive mechanism, enabling *P. australis* to withstand accelerating environmental changes and to successfully colonize and persist in estuarine habitats characterized by fluctuating salinity.

### 4.3 Patterns of DNA Methylation Stability in *P. australis* Across Generations

*P. australis* exhibited pronounced genotype‐specific variation in natural environments, with CHG hemi‐methylation fluctuating across generations (Figure 4). DNA methylation patterns are not stably preserved during individual plant development, even within identical environments, as cytosine methylation may be randomly acquired or lost, causing spontaneous epistatic mutations (Vanden Broeck et al. 2024). This observed phenomenon is consistent with the spontaneous epistatic mutations in the distantly related species *T. arvense* across three consecutive generations grown in natural environments. Although the DNA methylation patterns are relatively conserved under controlled environments, the methylation status of some gene loci can also change due to random fluctuations across generations and minor environmental disturbances (Geng et al. 2020).

Notably, the DNA methylation patterns of *P. australis* were partially inherited in the offspring under successive salt stress, with CG methylation levels consistently decreasing and CHG hemi‐methylation fluctuating across generations (Figure 4). This epigenetic flexibility confers plasticity for short‐term adaptation. At the same time, the genetic background plays a decisive role in constraining epigenetic variation, resulting in genotype‐specific stability (Alvarez et al. 2021; Lin et al. 2022). For instance, genotype G10 exhibited cumulative generational effects, potentially functioning as a stress memory mechanism that enhances response robustness. Such differences among genotypes in maintaining or accumulating methylation changes likely reflect variation in the regulation of DNA methyltransferase or demethylase activity (Bewick and Schmitz 2017; Zhang and Zhu 2024).

However, the specific genetic determinants driving these epigenetic variations remain unresolved, highlighting the need for future studies to identify the precise genetic loci regulating epigenetic stability. Our findings reveal substantial epigenetic responses and adaptive regulation under long‐term transgenerational salt stress in *P. australis* , suggesting that genotypes with higher epigenetic plasticity are promising candidates for ecological restoration in saline wetlands. This underscores the importance of integrating molecular‐level stress memory into germplasm screening strategies to better predict plant resilience under future environmental changes.

## 5 Conclusions

In summary, this study demonstrates that the genotype, as well as parental and offspring salinity environments, can jointly influence plant phenotypic responses and adaptive strategies, in which epigenetic and metabolic pathways may be involved in complex regulatory mechanisms. Salt stress significantly reduced *P. australis* growth, with variable phenotypic responses observed across genotypes. However, parental salt stress exposure could alter DNA methylation patterns and be associated with phenotypic traits in the offspring of different genotypes. Particularly, it enhanced adaptive transgenerational plasticity after exposure to the same salt stress in the parental and offspring generations. The study also demonstrated that continuous exposure to saline environments can consistently induce cumulative effects on epigenetic patterns, leading to long‐term epigenetic regulation in plants. In addition, parental salt stress experiences also alter DEGs in a specific genotype when offspring re‐experience salt stress, causing enrichment of key genes in antioxidant, metal ion binding, and secondary metabolic pathways, thereby affecting offspring salt tolerance. Together, these findings advance our understanding of stress adaptation in wetland plants, and suggest that transgenerational epigenetic plasticity may contribute to ecological success under persistent environmental stress.

Due to the slow establishment of the offspring, different generations in this study were conducted in different seasons. Temporal variation in environmental factors such as temperature and photoperiod may therefore have partly influenced plant performance across generations. In addition, given the relatively large number of samples, MSAP was used to characterize methylation patterns. Considering the limitations of this approach and limited replication, further research employing high‐resolution bisulfite sequencing with larger sample sizes will be essential to precisely identify the specific genomic loci and regulatory mechanisms driving these transgenerational effects.

## Author Contributions

**Yu‐Han Chen:** conceptualization (equal), data curation (lead), formal analysis (lead), investigation (lead), methodology (equal), visualization (lead), writing – original draft (lead), writing – review and editing (equal). **Chun‐Lin Wang:** data curation (equal), writing – original draft (equal). **Jian‐Qiao Meng:** data curation (equal), investigation (equal). **Yi‐Fan Liu:** data curation (equal). **Tao Fang:** data curation (equal). **Yao‐Jun Zhu:** writing – review and editing (equal). **Fang‐Li Luo:** conceptualization (lead), funding acquisition (lead), methodology (equal), supervision (lead), writing – review and editing (lead).

## Funding

This work was supported by the National Natural Science Foundation of China, 32371584, 32071525.

## Conflicts of Interest

The authors declare no conflicts of interest.

## Supporting information


**Table S1:** Effects of different *Phragmites australis* genotypes, generations, and their interactions on the phenotype and DNA methylation levels under control and salinity environments across three successive generations.
**Figure S1:** Neighbor‐joining tree of 416 *P. australis* individuals from Liaohe, Yellow River, Yangtze, and Minjiang River Estuaries in China.
**Figure S2:** Effect size of different *P. australis* genotypes on parental traits under salt stress. The effect values were calculated as the increase (positive) or decrease (negative) in the salt treatment relative to the control. Significance levels: ***p < 0.001, **p < 0.01, *p < 0.05.
**Figure S3:** Gene Ontology functional annotation (A) and Kyoto Encyclopedia of Genes and Genomes pathway enrichment analysis (B) of differentially expressed genes in G4 plants obtained from the comparison between parental salt stress treatment and parental control treatment under offspring salt stress.

## Data Availability

The data supporting this study's findings are available on https://doi.org/10.6084/m9.figshare.28710704.
