# Supplementary material for: Inheritable Epigenetic Memory Induced by Parental Salt Stress Influences Transgenerational Plasticity of Phragmites australis
Source: Ecol Evol. 2026 Mar 30;16(4):e73343. doi: 10.1002/ece3.73343 (PMC13107263; doi:10.1002/ece3.73343)
Supplement: Supplementary file 1 — Table S1: Effects of different Phragmites australis genotypes, generations, and their interactions on the phenotype and DNA methylation levels under control and salinity environments across three successive generations. Figure S1: Neighbor‐joining tree of 416 P. australis individuals from Liaohe, Yellow River, Yangtze, and Minjiang River Estuaries in China. Figure S2: Effect size of different P. australis genotypes on parental traits under salt stress. The effect values were calculated as the increase (positive) or decrease (negative) in the salt treatment relative to the control. Significance levels: ***p < 0.001, **p < 0.01, *p < 0.05. Figure S3: Gene Ontology functional annotation (A) and Kyoto Encyclopedia of Genes and Genomes pathway enrichment analysis (B) of differentially expressed genes in G4 plants obtained from the comparison between parental salt stress treatment and parental control treatment under offspring salt stress. [file ECE3-16-e73343-s001.docx]

**Support Information**

**Table S1** Effects of different *Phragmites australis* genotypes, generations, and their interactions on the phenotype and DNA methylation levels under control and salinity environments across three successive generations.

|  | Traits | Genotype | Generation | Genotype × Generation |
| --- | --- | --- | --- | --- |
| Control | CHG hemi-methylation | 1.78 | **6.44^**^** | **2.20^**^** |
|  | CG methylation | **2.51^**^** | **8.25^***^** | **1.85^*^** |
| Salinity | CHG hemi-methylation | 0.65 | **4.74^*^** | 1.51 |
|  | CG methylation | **2.08^*^** | **5.60^**^** | **2.41^**^** |

*F* values are provided. Appropriate denominator degrees of freedom (*df*) were determined by Satterthwaite's approximation. The *df* for genotype effects, generation effects, and their interactions were approximately (12, 111), (2, 111), and (24, 111), respectively. Significant effects are in bold with *p*-values as follows: ^***^*p* < 0.001, ^**^0.001 ≤ *p* < 0.01, and ^*^0.01 ≤ *p* < 0.05.


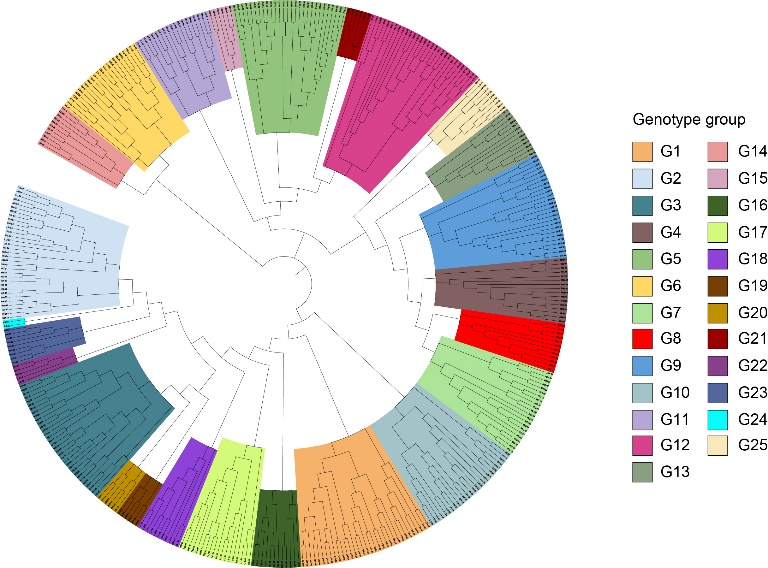


**Figure S1** Neighbor-joining tree of 416 *P. australis* individuals from Liaohe, Yellow River, Yangtze, and Minjiang River Estuaries in China.

**
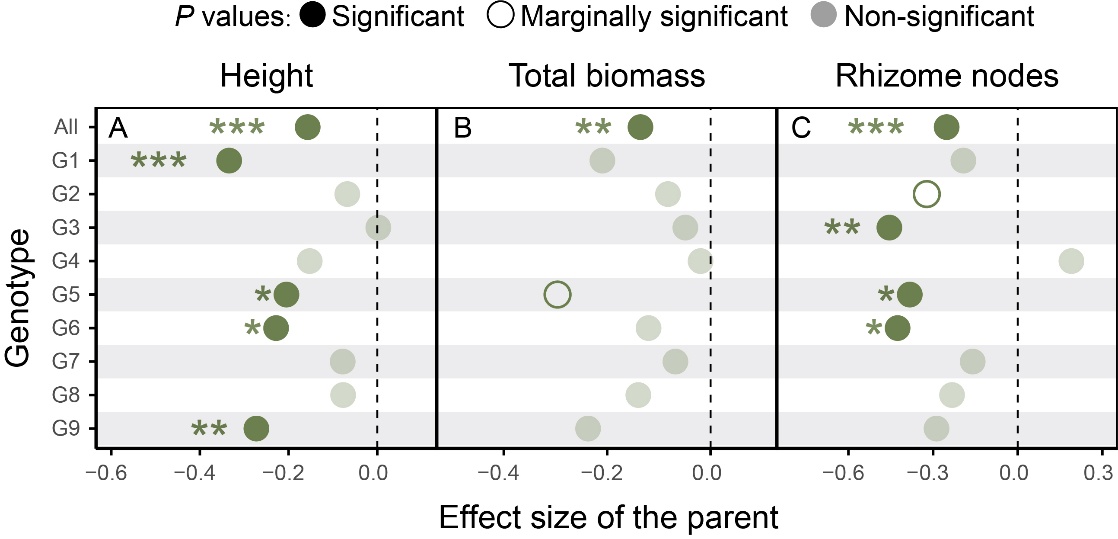
**

**Figure S2** Effect size of different *P. australis* genotypes on parental traits under salt stress. The effect values were calculated as the increase (positive) or decrease (negative) in the salt treatment relative to the control. Significance levels: ^***^ *p* < 0.001, ^**^ *p* < 0.01, ^*^ *p* < 0.05.

**
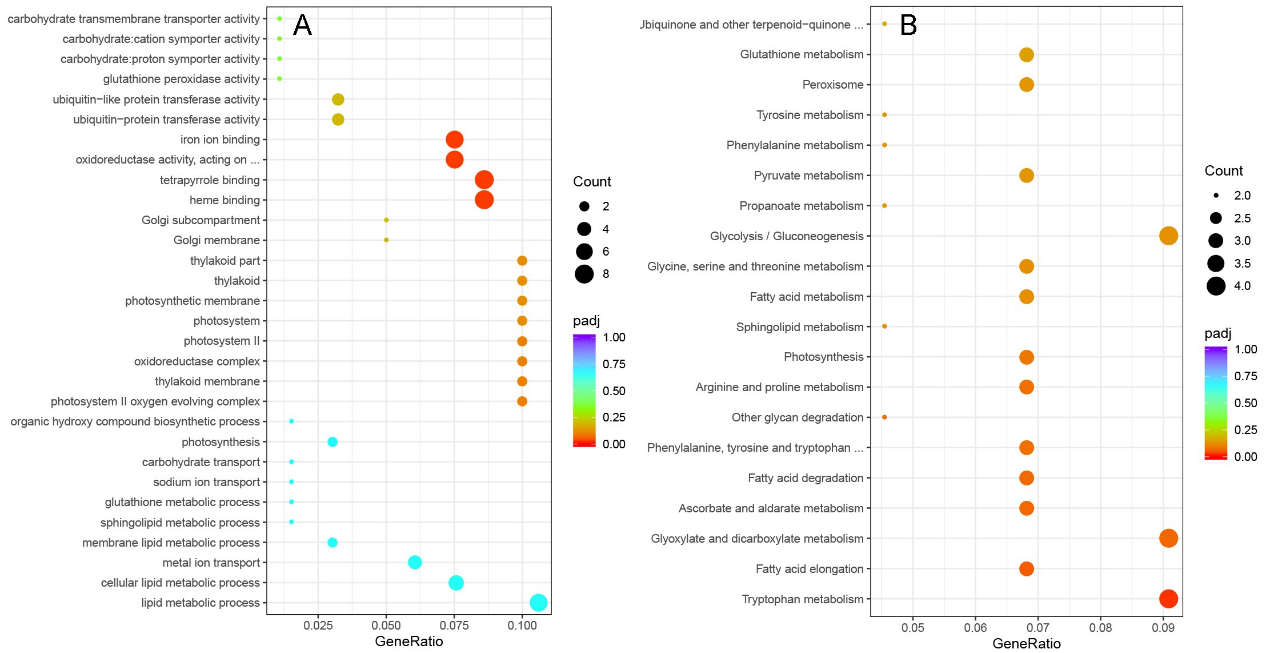
**

**Figure S3** Gene Ontology functional annotation (A) and Kyoto Encyclopedia of Genes and Genomes pathway enrichment analysis (B) of differentially expressed genes in G4 plants obtained from the comparison between parental salt stress treatment and parental control treatment under offspring salt stress.
